# The Impact of Predation by Marine Mammals on Patagonian Toothfish Longline Fisheries

**DOI:** 10.1371/journal.pone.0118113

**Published:** 2015-03-04

**Authors:** Marta Söffker, Phil Trathan, James Clark, Martin A. Collins, Mark Belchier, Robert Scott

**Affiliations:** 1 Centre for Environment, Fisheries & Aquaculture Science; Pakefield Rd, Lowestoft, United Kingdom; 2 British Antarctic Survey; High Cross, Madingley Road, Cambridge, United Kingdom; 3 Marine Resources Assessment Group; 18 Queen Street, London, United Kingdom; 4 Government of South Georgia and the South Sandwich Islands; Ross Road, Stanley, Falkland Islands; Point Blue Conservation Science, UNITED STATES

## Abstract

Predatory interaction of marine mammals with longline fisheries is observed globally, leading to partial or complete loss of the catch and in some parts of the world to considerable financial loss. Depredation can also create additional unrecorded fishing mortality of a stock and has the potential to introduce bias to stock assessments. Here we aim to characterise depredation in the Patagonian toothfish (*Dissostichus eleginoides*) fishery around South Georgia focusing on the spatio-temporal component of these interactions. Antarctic fur seals (*Arctocephalus gazella*), sperm whales (*Physeter macrocephalus*), and orcas (*Orcinus orca*) frequently feed on fish hooked on longlines around South Georgia. A third of longlines encounter sperm whales, but loss of catch due to sperm whales is insignificant when compared to that due to orcas, which interact with only 5% of longlines but can take more than half of the catch in some cases. Orca depredation around South Georgia is spatially limited and focused in areas of putative migration routes, and the impact is compounded as a result of the fishery also concentrating in those areas at those times. Understanding the seasonal behaviour of orcas and the spatial and temporal distribution of “depredation hot spots” can reduce marine mammal interactions, will improve assessment and management of the stock and contribute to increased operational efficiency of the fishery. Such information is valuable in the effort to resolve the human-mammal conflict for resources.

## Introduction

Since the modernisation of longline fishery technology in the 1950s, incidents of depredation (fish removed from the gear by predators during hauling) of longline fisheries by toothed whales have been reported with increasing frequency and are now a global occurrence. Depredation by toothed whales can lead to considerable loss of the catch in some parts of the world [1] and can have implications for the economic viability of longline fisheries, the target stock assessment and management, as well as the status of the toothed whale populations themselves [2]. In tropical and sub-tropical waters, depredation is mainly due to false killer whales (*Pseudorca crassidens*) and short-finned pilot whales (*Globicephala macrorhynchus*) [2–4]. Longlines in colder waters are generally depredated by the true killer whales or orcas (*Orcinus orca*) and sperm whales (*Physeter macrocephalus*) [5–12].

Orcas and sperm whales in sub-Antarctic waters of the Southern Ocean interact mostly with longlines set for Patagonian toothfish (*Dissostichus eleginoides*, hereafter toothfish), the principle finfish fishery in this area and the remaining Southern Ocean (together with *Dissostichus mawsoni*). Longlines for toothfish in the Southern Ocean are set at depths outside the normal foraging range of many piscivorous marine mammals, typically below 1000 m, and are usually weighted or anchored to the sea bed. Depredation occurs mostly during hauling, when the catch becomes accessible in the surface waters. In particular orcas and sperm whales, but also Antarctic fur seals (*Arctocephalus gazella*, hereafter fur seals), are frequently seen feeding at the longlines during hauling [9, 11]. Orcas are reported to take a large proportion of the catch [10, 13]. Anecdotally they have also been observed to follow fishing vessels between sets and to feed selectively on toothfish, ignoring by-caught skates and grenadier. Loss of fish through depredation can cause financial loss to the toothfish fishing industry [11] and if not considered appropriately may lead to a bias in stock assessments through the underestimation of total mortality resulting from fishing and depredation combined (see also [9]).

Orcas in the Southern Ocean have repeatedly been identified as among the main species responsible for toothfish depredation [5–6, 8–11]. They are biologically divided into three or four subgroups, or ecotypes [14–16], each with distinctive prey choice and hunting behaviour. The large ecotype A orcas are found mainly in open waters and feed mostly on Antarctic minke whales (*Balaenoptera bonaerensis*). These orcas hunt in coordinated packs often moving over long distances. The B ecotype occurs mostly among pack ice and feeds on seals, especially Weddell seals [14]. A smaller type (tentatively classified as) B has recently been described, observed feeding on penguins [17]. Type B orcas are highly innovative in their hunting strategies [14] and are able to rapidly learn new behaviours, or at least are observed to display behaviour not previously recorded. The third ecotype C is found mostly in the Ross Sea, deep in the pack ice, and specialises in feeding on fish such as the Antarctic toothfish [18]. Finally, the type D orca has been sighted regularly since 2003 around Crozet Island, but has otherwise been seen only occasionally and very little is known about its biology (see [17] for more detailed descriptions of all ecotypes). The ecotypes associated with depredation in toothfish longline fishery have so far been identified as type A-like and type D around Crozet Islands [19], and possibly type B around South Georgia [10].

### Mitigation measures

Several measures have been developed and tested over the years to mitigate depredation by toothed whales (reviewed in [1–2]). For orcas these include devices such as acoustic harassment devices (AHDs) which emit a deterring sound, the physical protection of the catch by nets, hooks or wires [2, 4, 7] or changes in fishing practise such as moving to a different area when orcas are present, changing offal dumping practises or using lines of different length [11, 13]; these mitigation measures are best developed through close involvement with fishing vessel operators [20]. The benefits of mitigation measures have been limited. AHDs have, to date, shown little effect when trialled in the Southern Ocean [13]. The use of physical deterrents (including nets to shroud the catch) has proved difficult to adapt in areas where the fishery collects data for stock assessment through tagging and releasing a proportion of its catch, as these measures can damage fish by abrasion and multiple hook damage, leaving them unsuitable for tagging. The most effective method yet to reduce depredation of catch is for a vessel to tie off its gear and move away when orcas arrive, and return to collect it later [13, 20]. However, the distance necessary to travel is substantial (>20–40 nm [11, 13]), and is an additional economic burden on the fishery in terms of fuel and time.

### Aims

In this paper, we set out to gain an understanding of where, when, and how much depredation occurs in the toothfish fishery around South Georgia by examining catch rates (catch per unit effort, CPUE), mammal abundance, movement, and mammal-vessel interactions. We describe new insights into mammal depredation behaviour around South Georgia in the South Atlantic and propose ways to mitigate depredation based on orca predatory behaviour.

## Methods

### Data

**Fig 1:**
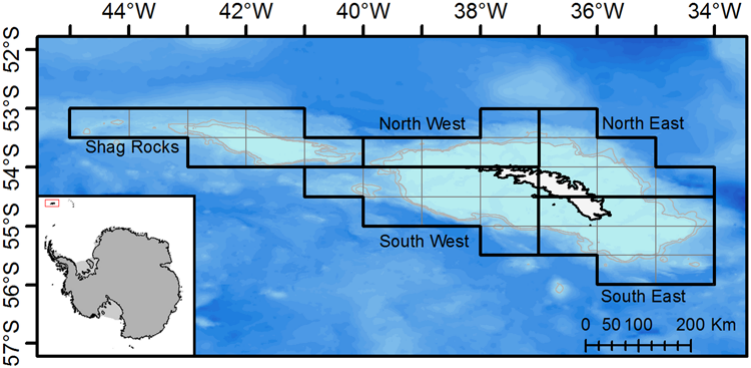
Geographic location of South Georgia. The area was sub-divided into 0.5x1 degree squares, which were grouped into 5 geographical areas: Shag Rocks, North West, North East, South West, and South East. The gray line denotes the 500m bathymetric line.

Data were obtained from the Commission for the Conservation of Antarctic Marine Resources data centre (CCAMLR; having been collected under the CCAMLR Scheme of International Scientific Observation) and from officially reported catch and effort statistics for the region corresponding to CCAMLR statistical subarea 48.3 (34–45°W, 53–56°S), in the southwest Atlantic (see Fig. 1). Between 1996 and 2012 data on catch, and associated observations, were collected from each fishing vessel by scientific observers, covering a total of 268 fishing cruises. Independent scientific fisheries observers are present on all commercial fishing boats in this area (according to the CCAMLR Scheme of International Scientific Observation). More than 87% of the longlines set were observed and mammal interactions with the catch were documented. In addition to the independent scientific observations, orca and sperm whale sightings by the vessel skippers were available for all sets during the years 2011 and 2012. The vessel observations allowed a cross validation with the data from observers, who do not observe every line. Historically, the vessels have operated for varying intervals across years. Since 1995, the fishery has been restricted to the winter months and since 2004 it has been further limited to between mid-April and mid-September. Two longline types are used, the Autoline-system and Spanish-type longlines with on average just over 7000 hooks per line (range 1000–36,000 hooks/line), deploying 1–3 lines per set and fishing between 200 and 2,000 m, generally at 700–1,600 m.

### Data preparation

Prior to analysis, the observations were screened for consistency across ships, years and fishing areas. Data from 1996, the first year of 100% scientific observer coverage, were excluded due to inconsistencies in the data. Furthermore, only data from observed longline sets were used and only the three most abundant mammal species were investigated: orca, Antarctic fur seal, and sperm whale. They were not only most abundant but their interactions with longlines were also several magnitudes higher than other marine mammals (cumulating to 89.3% of total data). For the purpose of estimating the spatial distribution of depredation, South Georgia was subdivided into five geographical areas (Fig. 1): Shag Rocks, North West Georgia, North East Georgia, South West Georgia, and South East Georgia. These areas were further subdivided into boxes of 0.5° latitude x 1° longitude for finer scale geospatial analysis.

To estimate seasonal movements and overall abundance for each species the number of observations in each box and each area was divided by the number of longline sets (observations per unit effort, OPUE), thus accounting for any bias in fishing location and frequency. The geospatial 0.5°x1° boxes were too large for higher resolution analysis such as spatial distribution of catch rates, depredation, and investigation of orca movements in relation to fishing vessels. For these analyses, average catch rates, number of longline sets and ships, and total sum of catch was calculated for 10x10 km squares, under scenarios of both orcas absent and orcas present

In order to determine whether orcas follow fishing vessels between sets, the dataset was investigated to establish whether it was physically feasible for observations on the same day to come from the same group of orcas. Firstly all orca observations with a unique date (singular observations per day) were discarded. Potential tracks were formed by finding succeeding observations within 50 km distance of each other on the same day, where observations further apart were assumed as part of an independent potential track. The limit distance of 50 km was based on approximate orca mean daily travel distances previously tracked in the Ross Sea [21].

To test whether orcas are attracted to the sound of vessels hauling their lines, we investigated the relationship of fishing intensity (longlines 10km^-2^ month^-1^) and number of orca interactions (orcas longline^-1^ 10km^-2^ month^-1^), as higher fishing intensity in an area is typically associated with higher rates of hauling activity and its associated sound. Therefore, lack of correlation between hauling activity and orca interactions would indicate that orcas are not primarily attracted by hauling activity into the area. If it were the case, the temporal movement of the fleet would be mimicked by the temporal movement of the orcas.

The annual catch rates were calculated under 21 different scenarios in each of the five geographical areas: no mammals present; orcas/fur seals/sperm whales, present/absent/feeding/not feeding; and combinations of the above (e.g. orca present + sperm whales absent + fur seal feeding).

### Statistical analysis

After initial data exploration, the best-fitting models for mammal abundance (mammal observations/area/longline set), mammal interactions (mammal observations in relation to other species), and mammal-vessel interactions (mammal observations in relation to fishery and vessel-related factors) were established using generalised linear mixed models (GLMM) or generalized linear models (GLM) where appropriate. Temporal, spatial and observer/vessel variables were used as random effects where fitting. Stepwise model simplification was obtained by removing insignificant terms one after the other. This produced the minimal adequate model. Tree models [22] were used alongside the mixed models to obtain a general understanding of interactions between the fishery and the three abundant mammal species [23]. GLMMs were fitted by maximum likelihood based on Laplace approximation. The final model was re-fitted under restricted maximum likelihood using the ‘lme4’ package [24]. The best-fitting model for catch effort per year was re-fitted with Penalised Quasi-Likelihood (GLMM-PQL) following the Tweedie distribution. A log-link was used as the data were not normally distributed and included many low value/zero observations, utilising both the ‘MASS’ and ‘tweedie’ packages and their dependencies [25–26]. A detailed description and methodology for this approach can be found in Candy (2004) and Shono (2008) [27–28]. All data were analysed with the statistical software R v. 2.15.2 [29] and all spatial analysis was carried out in ESRI ArcGIS 9.3 (ESRI, Redlands, CA) or R v 2.15.2.

## Results

The majority of sets did not encounter any mammals (Table 1). 25% of the observed marine mammals were sperm whales, making them the most frequently observed mammals at longlines in recent years. Until around 2009 Antarctic fur seals were the most abundant (Table 1). Orcas were only observed at 4.7% on average of longline sets per year. Whale sightings reported by the observers *versus* those reported by skippers were largely consistent (F _1, 1748_ = 1.941 e^04^, adj R^2^ = 0.92, p < 2.2 e-^16^ for orcas, F _1, 2354_ = 2.411 e^04^, adj R^2^ = 0.91, p < 2.2 e-^16^ for sperm whales); on average skippers saw 0.3 (median 0) more orcas and 0.6 (median 0) more sperm whales per longline set in 2011 and 2012. Orcas were usually seen in groups of 8–10 animals, while sperm whales were mostly seen in pairs.

**Table 1:** Number of observed longline sets interacting with mammals between 1997 and 2012 around South Georgia.

	Sets with observations				Number of mammals			% of sets observing mammals			Mammal ratio per set		
Year	*Antarctic fur seal*	*sperm whale*	*orca*	*No mammals*	*Antarctic fur seal*	*sperm whale*	*orca*	*Antarctic fur seal*	*sperm whale*	*orca*	*Antarctic fur seal*	*sperm whale*	*orca*
1997	159	371	84	662	1493	765	669	11.24	26.24	5.94	1.06	0.54	0.47
1998	71	470	102	806	2168	1067	1008	4.73	31.31	6.80	1.44	0.71	0.67
1999	197	761	123	728	3822	1938	1347	10.34	39.93	6.45	2.01	1.02	0.71
2000	105	592	107	1124	3834	1384	778	5.14	28.99	5.24	1.88	0.68	0.38
2001	94	409	54	1085	237	615	85	4.66	20.28	2.68	0.12	0.30	0.04
2002	268	678	132	1842	2242	1295	1028	8.86	22.41	4.36	0.74	0.43	0.34
2003	300	1297	161	2637	5832	2466	1481	6.37	27.53	3.42	1.24	0.52	0.31
2004	245	652	89	1794	1467	1285	1076	8.06	21.46	2.93	0.48	0.42	0.35
2005	338	574	110	1018	1317	1338	645	15.64	26.56	5.09	0.61	0.62	0.30
2006	526	668	119	1218	2891	2378	971	20.45	25.97	4.63	1.12	0.92	0.38
2007	253	452	95	1425	1475	1087	903	11.34	20.25	4.26	0.66	0.49	0.40
2008	136	549	121	2065	979	1164	967	4.65	18.79	4.14	0.34	0.40	0.33
2009	620	634	107	1530	2879	1665	822	20.69	21.16	3.57	0.96	0.56	0.27
2010	65	512	138	1453	301	1394	842	2.96	23.35	6.29	0.14	0.64	0.38
2011	90	283	69	959	134	671	372	6.28	19.74	4.81	0.09	0.47	0.26
2012	17	518	77	912	35	1558	568	1.11	33.72	5.01	0.02	1.01	0.37
**average**	217.75	588.75	105.50	1328.63	1944.13	1379.38	847.63	8.91	25.48	4.73	0.81	0.61	0.37
**total**	3484	9420	1688	21258	31106	22070	13562						

On average, 4.7% of sets interact with orcas, 8.9% interact with fur seals and 25.4% interact with sperm whales.

### South Georgia depredation

The three most important factors affecting catch rates of longline vessels were the (i) year (GLMM, F = 939.03, p<0.0001), with a clear split between years 1997–2008 and 2009 to present (F = 6.37, p = 0.0116), (ii) the nationality of the vessel (p<0.0001), and (iii) orca presence (F = 102.54, p<0.0001). Before 2009, orcas were the third most important factor affecting catch rates, and their impact varied with location; after 2009, catch rates differed only between the north and the south side of the island.

**Fig 2:**
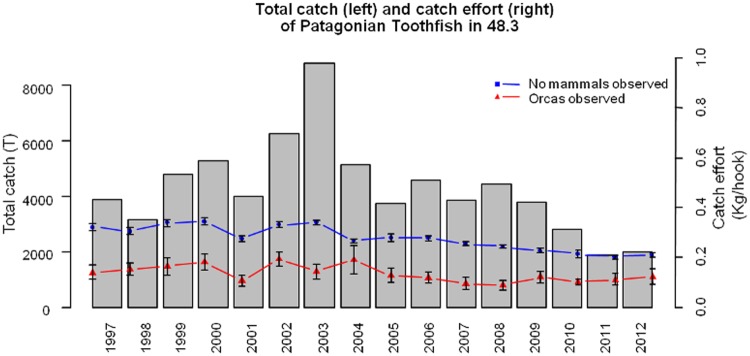
Historic catch rates comparison and total catch of Patagonian toothfish. Gray bars show total catch of toothfish, blue line shows catch rates without mammals at the longline set, and red line shows catch rates when orcas were observed at the longline set.

The catch per unit effort (CPUE, kg/hook) of toothfish was consistently higher with no mammals seen near the lines, than when orcas were seen near the lines (Fig. 2, F_3, 30568_ = 621.2, p = 0.00001). This difference was attributed directly to orcas feeding on the catch of the longline, with supplementary evidence for feeding provided by photographic identification of feeding orcas. Lines where orcas were photographed and identified while feeding had significantly lower catch rates (between 63% and 97% lower; two-sample t-test, t_8_ = -5.23, p = 0.0008) than the spatially and temporally nearest unaffected longline. The highest number of orca OPUE (observations/area/line) averaged across all years is in the North East (0.88 ± 0.11), followed by Shag Rocks (0.79 ± 0.10), North West (0.67 ± 0.11), South East (0.44 ± 0.08) and lastly South West (0.13 ±0.05).

**Fig 3:**
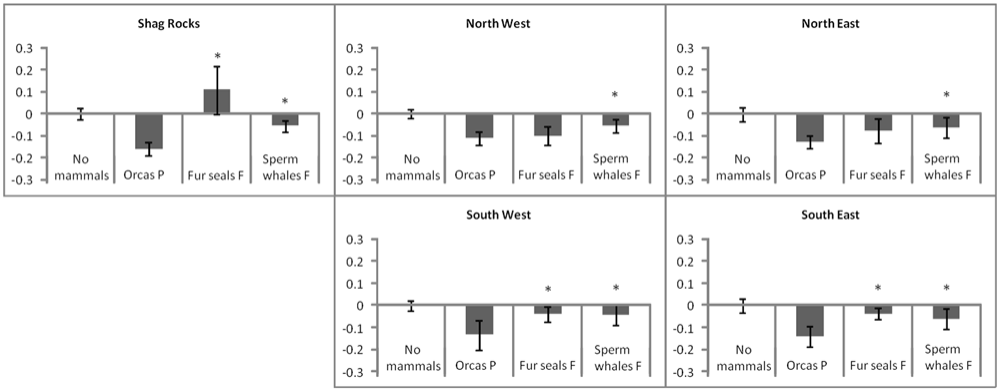
Difference in catch rates under various depredation scenarios. Mean difference (Tukey’s honest statistical difference) in catch rates of the three main mammal scenarios to the no-mammal scenario around South Georgia. P—present, F—feeding. Originally 21 scenarios were calculated (orcas, sperm whales, fur seals: present/ absent, feeding/not feeding, and combinations of these) and the three most damaging scenarios to catch rates were chosen to be graphically represented. No-mammal-scenario average catch rates: Shag rocks = 300±0.06 g, North West = 233±0.05 g, North East = 276±0.07 g, South West = 253±0.05 g, and South East = 272±0.08 g of toothfish/hook. Stars denote difference between mammal scenarios.

The catch rates were significantly lower in all scenarios where orcas were present (see above; Fig. 3), and the difference between depredation by orcas and sperm whales or fur seals was largest on the south side (Anova; F_3,3670_ = 11.45, p = 0.02 (sperm whales) and p = 0.008 (fur seals) in the South West, F_3,3881_ = 26.91, p = 0.008 (sperm whales) and p < 0.0001 (fur seals) in the South East) and around Shag Rocks (Anova; F_3,8311_ = 77.9, p < 0.0001 for both). On the north side of South Georgia, there was no difference between orca and fur seal depredation (Anova; F_3, 3073_ = 43.4, p = 0.92 in the North West and F_3,4942_ = 50.4, p = 0.18 in the North East), but there was a difference between orca and sperm whale depredation (p = 0.003 in the North West and p = 0.02 in the North East). Therefore, orca depredation significantly lowered catch rates, and the depredation by fur seals and sperm whales differed between the north and south side of South Georgia.

### Spatial and temporal distribution of orca depredation

**Fig 4:**
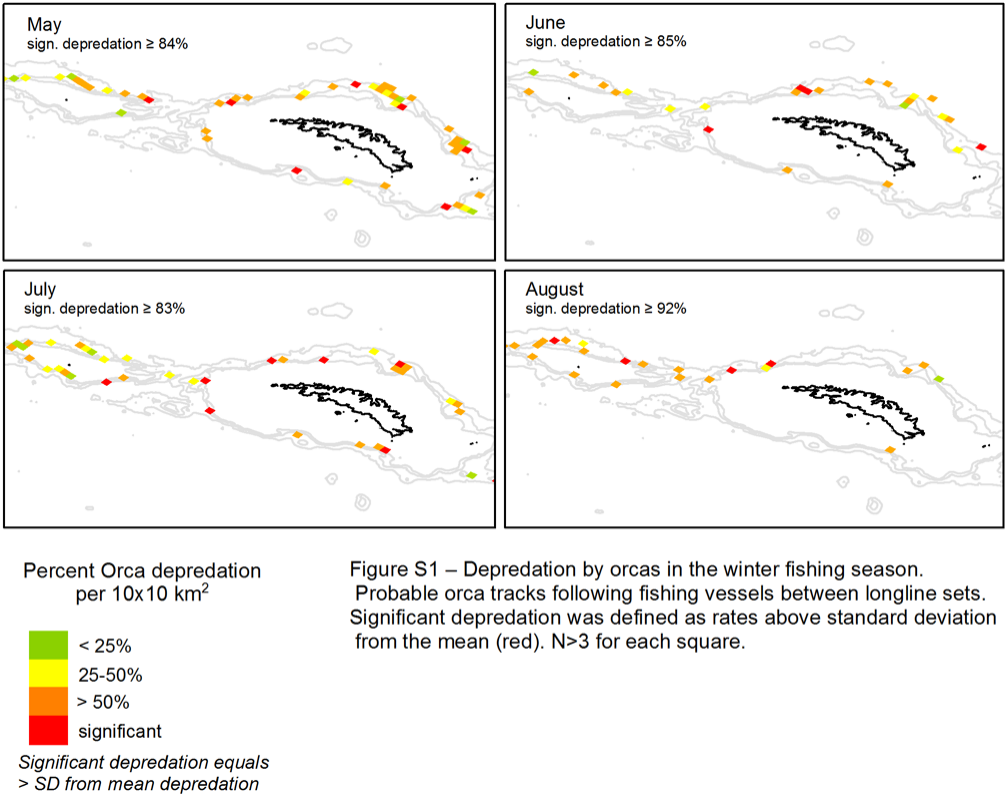
Depredation by orcas in the winter fishing season. Percent depredation (% CPUE difference between depredated and non-depredated lines) per 10 km^2^ for each month of the winter fishing season. Significant depredation was defined as rates above standard deviation from the mean (red). Each square represents 10 km^2^. N>3 for each square.

The CPUE GLMM demonstrated that both catch rates and orca depredation varied spatially. Fig. 4 shows the percentage of depredation loss for affected longlines within each 10km^2^. This illustrates that depredation occurred across all areas, but with ‘hot spots’ where depredation is high. Most interactions with orcas around South Georgia were single encounters. However, potential tracks of vessels by orcas were located in those same ‘hot spots’ (S1 Fig). When lines were depredated the loss was typically at least 25% of the catch, with the majority of affected sets losing 50% or more of their initial take (Fig. 4). High losses typically occurred on the north side of South Georgia in May and June, and around Shag Rocks (to the west of South Georgia) in July and August.

### Attraction of orcas by hauling activity

We found no correlation on the comparatively coarse scale on which we analyzed the data between fishing intensity and orca interactions for any given month (May p = 0.08, June p = 0.39, July p = 0.48, August p = 0.89). However, on a localised scale, orcas do seem to follow ships, mostly in May and June in the North West, north of Cumberland Bay, and around Shag Rocks (S1 Fig). These relatively small areas experience significant losses of catch rates due to orca depredation in May and June.

### Mammal distribution around South Georgia

**Fig 5:**
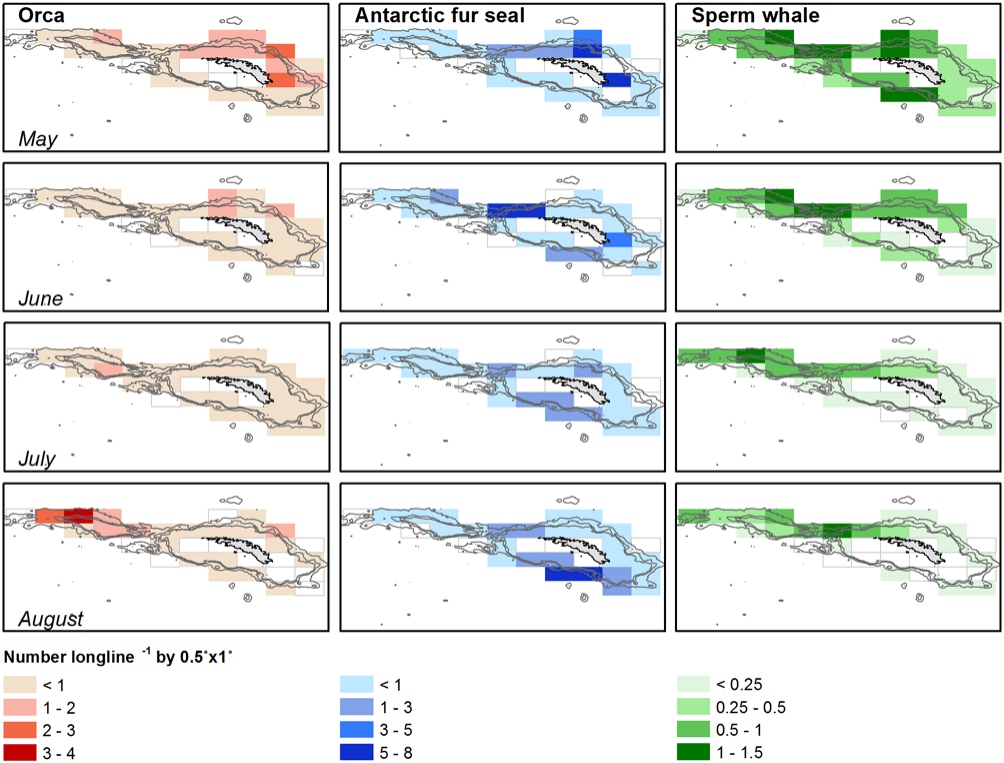
Mammal movements over the winter season. Observations per unit effort (animals/longline/box) of orcas, fur seals and sperm whales around South Georgia throughout the winter season.

Seasonal movement of the orcas (Fig. 5, S1 Table) around the island appeared to be driven by factors other than the attraction to fishing vessels. In some areas, orcas were more frequently seen than in others (logLik dif = 10.61; p = 0.027); Shag Rocks had significantly more orca observations (p<0.001), while significantly fewer were seen in the South West (p = 0.001). The orcas concentrated on the north side of the island in May and throughout June, and then slowly moved towards Shag Rocks where they aggregated north-west of Shag Rocks in August (logLik dif = 11.89, p = 0.01). Individual orcas, identified morphometrically, were seen repeatedly and in different years. Their movements from the north/east in autumn to the west in winter concurred with the generally observed seasonal movement pattern (Fig. 5).

The spatial distribution of fur seals (OUPE) around the island is dependent on factors such as area, month, year, and interactions between these factors (S1 Table). A general annual pattern of distribution showed small aggregations of fur seals on the north side of South Georgia in April at the same time when orcas aggregate here (logLik dif = 6.94, p = 0.024, Fig. 5). Larger groups were seen around the South West in late July and August, but this pattern is variable between years (logLik dif = 6.91, p = 0.011). However, the model describing this pattern fits relatively poorly, thus indicating that it does not capture those factors adequately which explain seal spatial and temporal distribution.

Sperm whales are seen more frequently to the west of the island in autumn (May/June) and at Shag Rocks in July and August (logLik dif = 14.97, p = 0.002, Fig. 5), when they aggregate in areas similar to the orcas. Their distribution around the island shows some annual variation (logLik dif = 13.27, p = 0.002). Sperm whales are more often seen in the North West andat Shag Rocks than in the other areas (logLik dif = 8.36, p = 0.005).

## Discussion

### Orca depredation and seasonal movement around South Georgia

The orcas around South Georgia have a detrimental impact on the toothfish longline landings as they can remove a significant proportion of the catch. However, interactions occur on a temporally and spatially restricted scale and only a small proportion of sets encounter orcas. Depredation rarely exceeds 5% of the total catch in the fishery to date. High orca depredation is concentrated in an 80km^2^ area north of Cumberland Bay and around Shag Rocks, and occurs mostly within May and August (see also [13]). These two ‘hot spots’ are also the areas where orcas are potentially following vessels between longline sets. There was no clear evidence that the sound of hauling attracted orcas, although a trend for aggregating in areas of high longline activity was seen in May. To avoid seabird by-catch, the South Georgia fishery only operates during the winter months, and vessels could potentially be subject to higher depredation during summer months, similar to depredation in the North Pacific [12]).

The movement of orcas around South Georgia does not appear to be driven by the toothfish fishery. The high presence of orcas and the high fishing intensity to the north of South Georgia in May suggests that orcas and the fishery are in the same area for perhaps the same reason: the high and predictable presence of krill to the north of South Georgia in late autumn/winter attracts fish [30]. Why orcas aggregate towards the northwest of Shag Rocks in August is still unknown. It occurs at the same time as when toothfish aggregate in the same area for spawning [31]. The north side of Shag Rocks is an important spawning ground of toothfish, and female toothfish of up to 100 cm length rise in some numbers in late July-August from deep waters to waters as shallow as 400–600 m [31]. Elsewhere in the world orcas have been observed to follow fish to spawning areas [32], so a similar scenario whereby the more easily accessible toothfish after spawning attracts orcas is possible; however, further research is necessary to answer this question.

The small spatial and temporal extent of depredation could be due to a small group of orcas north of Cumberland Bay specialising in depredation. Similar observations were made at Crozet Island where a small number of pods are responsible for over 80% of depredation [11]. However, further investigation including identification of individual orcas and their tagging is needed to confirm or reject this hypothesis for South Georgia. Small individual pods of orcas depredating on toothfish would be able to sustain themselves by depredation, however, if the number of pods living solely from depredation increases it will become increasingly difficult. If all known orcas around South Georgia were to rely on depredation, they would currently be able to cover only 20–70% of their energetic expenses. The difference in weight of toothfish catches between depredated and non-depredated lines was in almost all cases more than 100 kg per orca (per depredated line), which would be sufficient to cover the estimated necessary 100 kg/day of toothfish for female and 124 kg/day for male orcas [33] (assuming 184 kcal/100g raw toothfish, seafood.org). Catch rates for any scenario with orcas present, whether observed feeding or not, were significantly lower than catch rates when no orcas were present. It suggests that when orcas were present, they fed off the longline, but the observer may not always have been able to identify feeding behaviour.

Photographed individuals depredating from the South Georgia longline fishery [10] fit the description of the small type B orca, also known as the Gerlache orca [17], which to date has mainly been observed around the Gerlache Strait feeding on penguins [34]. The pattern of orca movement around South Georgia and their interactions with the longline fishery suggests that fish is an important nutritional resource for this orca ecotype. It is suspected that its main diet component actually consists of fish [17], although direct observations have not been made to date. Repeated observations of the same orcas in different years moving east to west suggests that some orcas at South Georgia are at least partially resident (a core group exists in autumn/winter) and the aggregation occurring at Shag Rocks in winter consists more likely of these residents than of orcas migrating from other areas. Very little is known about the annual movement or possible migrations of the small type B. The large type B orcas travel locally up to 50 km per day within a limited range [21], but also make long seasonal return trips towards South America of up to ~ 9400 km [35]. It is unclear whether the small type B ecotype has similar movement patterns (with possible short trips to South America), or whether they stay at South Georgia for the entire year. The large type B have been observed in the Antarctic both in summer and in winter [14].

The inventive prey acquisition methods and behavioural adaptability displayed by orcas (e.g. [14, 20]), especially of ecotype B, make solving this conflict-of-interests between fishery and depredators a challenging task. Skippers already report mothers bringing their calves to the longlines for depredation. In the case of the South Georgia longline toothfish fishery the knowledge of where and when significant depredation can be expected will be valuable for reducing catch losses. Orcas are often found near those areas that offer the best catch rate [10, 12–13]. Fishing in less profitable but orca-free areas could be a trade off worth considering for a reduction or avoidance of depredation.

### Fur seal and sperm whale seasonal movements

In contrast to orcas, neither sperm whales nor fur seals were significant contributors to depredation. Around Shag Rocks, toothfish catch rates were higher in the presence of fur seals. This may be because of higher productivity around Shag Rocks, including for fur seal and toothfish prey [9], which include the yellow-finned notothen (*Patagonothen guntheri*) around Shag Rocks [36], myctophids and squid [9]. Fur seals occur in May at locations where both the toothfish fishery and the orcas are present, and in the same areas where Antarctic krill (*Euphausia superba*) catch rates are high [30, 37]. Although the fur seals have been seen to feed off the longlines around South Georgia, especially on the north slope, krill constitutes the main part of their diet [38]; their concentration west and then south of South Georgia follows where krill is most abundant (e.g. north in May and June, south in August [39]).

The poor model fit indicates that the main factors in describing seal presence have not been captured, and including krill seasonality could prove to be a key explanatory component. Fur seal interactions with longlines were particularly high in 2009—a year when krill abundance around South Georgia was exceptionally low with large areas devoid of krill. The low interactions with longlines in 2012 coincided with the third-highest total catch of krill in the past 10 years (>55000 T for South Georgia) which suggests that krill was abundant enough to feed the seals, unlike in 2009.

Historically, interactions between sperm whales and the longline fishery were increasing around South Georgia [10]. Our current study showed that interactions have stabilised at around 20% of sets until 2012, when interactions with sperm whales suddenly increased to > 30% of longline sets. Despite the increase of encounters with sperm whales in 2012 catch loss due to sperm whale depredation did not increase in 2012. Increased interactions, however, means that avoidance manoeuvres by longliners need to be carried out more frequently, which increases costs even if catch rates remain the same (see also [10]).

Sperm whales do not follow longliners around South Georgia. They are seen more frequently in areas and at times when fur seals are seen less frequently—which may reflect their different habitat and prey preferences. Very little is known about the sperm whales around South Georgia, and since the end of commercial whaling their feeding habits around South Georgia have no longer been studied. Nevertheless, we know that they mainly feed on cephalopods [40] and to a large extent on the colossal squid *Mesonychoteuthis hamiltoni* [41–42], and also on Antarctic toothfish (*D*. *mawsoni*) [43]. Whether they follow a particular prey over the season is not possible to tell, but tagged sperm whales in Alaska involved in depredation were shown to follow the slope and depredate along it during their migration; some slowed their movement at a known high production area that included high abundances of prey squid [44]. Whether a similar situation is in place at South Georgia remains as work for the future.

The detailed analysis of mammal depredation has led to a better understanding of important factors affecting vessel CPUE and, by including spatial and temporal factors, has improved the depredation rate estimates used in the stock assessment. Understanding what behaviours underlie and drive depredation will help in the design of spatial management measures that could reduce or break the conflict cycle. In the case of the South Georgia orcas, the fishery and the orcas interact intensely over a short period of time in a restricted area; therefore, avoiding high-risk areas and times is likely to reduce depredation at least in the short term. It remains to be seen if avoidance of high-risk areas will lead to adaptive learning and a new distribution of orca depredation in the long-term.

## Supporting Information

S1 Table ANOVA statistics table for mammal interaction GLMM. (DOCX)

S1 Fig Depredation by orcas in the winter fishing season. Probable orca tracks following fishing vessels between longline sets.
(TIF)
