# Supplementary material for: The Impact of Predation by Marine Mammals on Patagonian Toothfish Longline Fisheries
Source: PLoS One. 2015 Mar 4;10(3):e0118113. doi: 10.1371/journal.pone.0118113 (PMC4349812; doi:10.1371/journal.pone.0118113)
Supplement: S1 Table — (DOCX) [file pone.0118113.s001.docx]

Table S1 - ANOVA statistics table for mammal interaction GLMM. A - Area, M – month, O – orca abundance, S – Antarctic fur seal abundance, Y – year; NumDF – numerator degrees of freedom, denDF – denominator degrees of freedom
