# Supplementary figures and images for: The Impact of Predation by Marine Mammals on Patagonian Toothfish Longline Fisheries
Source: PLoS One. 2015 Mar 4;10(3):e0118113. doi: 10.1371/journal.pone.0118113 (PMC4349812; doi:10.1371/journal.pone.0118113)

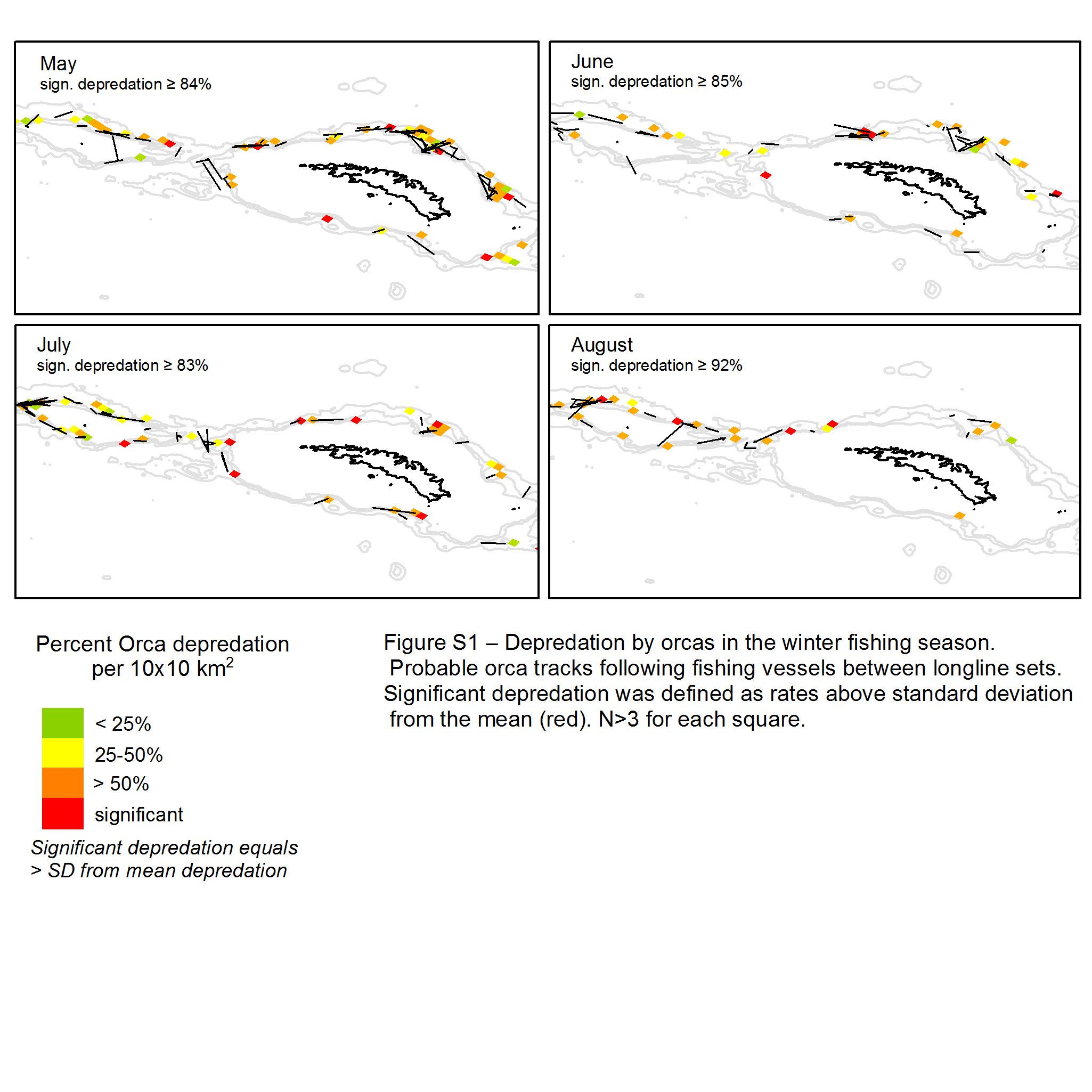

Supplement: S1 Fig — Probable orca tracks following fishing vessels between longline sets. (TIF) [file pone.0118113.s002.tif]
